# Modulation of spin-torque ferromagnetic resonance with a nanometer-thick platinum by ionic gating

**DOI:** 10.1038/s41598-021-01310-6

**Published:** 2021-11-05

**Authors:** Ryo Ohshima, Yuto Kohsaka, Yuichiro Ando, Teruya Shinjo, Masashi Shiraishi

**Affiliations:** Department of Electronic Science and Engineering, Kyoto University, Nishikyo-ku, Kyoto, 615-8510 Japan

**Keywords:** Magnetic properties and materials, Spintronics

## Abstract

The spin Hall effect (SHE) and inverse spin Hall effect (ISHE) have played central roles in modern condensed matter physics especially in spintronics and spin-orbitronics, and much effort has been paid to fundamental and application-oriented research towards the discovery of novel spin–orbit physics and the creation of novel spintronic devices. However, studies on gate-tunability of such spintronics devices have been limited, because most of them are made of metallic materials, where the high bulk carrier densities hinder the tuning of physical properties by gating. Here, we show an experimental demonstration of the gate-tunable spin–orbit torque in Pt/Ni_80_Fe_20_ (Py) devices by controlling the SHE using nanometer-thick Pt with low carrier densities and ionic gating. The Gilbert damping parameter of Py and the spin-memory loss at the Pt/Py interface were modulated by ionic gating to Pt, which are compelling results for the successful tuning of spin–orbit interaction in Pt.

## Introduction

Reciprocity has played significant roles in a wide variety of condensed matter physics. Indeed, the Seebeck and Pertier effects are interconnected under reciprocity, where the Seebeck effect allows charge flow by thermal gradient and the Pertier effect enables thermal flow by charge motion. Microscopically, the second order transport coefficient, *L*_ij_, for the Seebeck effect is equal to that of the Pertier effect, *L*_ji_, which is called Onsager reciprocity. A typical example of Onsager reciprocity in spintronics is spin-charge interconversion occurring in the spin Hall and inverse spin Hall effects. The spin Hall effect (SHE) allows conversion from a charge current to a spin current (a flow of spin angular momentum)^1–4^, and its reciprocal effect, the inverse spin Hall effect (ISHE), enables conversion from a spin current to a charge current^5^. Further examples of reciprocity can also be found in the fields of electromagnetism and electric circuit theory.

Spin–orbit torque (SOT) is now receiving significant attention in spintronics and spin-orbitronics^6–8^. SOT facilitates novel spintronic memory devices with high endurance and low power consumption. Magnetization reversal without damaging the tunnelling barrier of these devices can be realized by using SOT because spin current does not induce a net charge flow. Hence, efficient and tunable spin current generation is now one of the most pivotal and significant research issues in SOT-based spintronics technologies, because efficient spin current generation is indispensable in reducing energy consumption and tunable spin current generation allows easy on/off switching for magnetization reversal in devices. As mentioned above, a spin current is interconverted with a charge current by the SHE and the ISHE, where the spin–orbit interaction (SOI) governs the interconversion. The SOI is, in principle, larger in heavier materials, such as Pt^9,10^, β-Ta^7^ and W^11^, and the SHE and ISHE become more salient in such heavy materials. Hence, research targeting these materials has been of great significance in spintronics and spin-orbitronics.

Recent discovery of the gate-tunable ISHE in Pt shows a novel perspective in spin-orbitronics because the spin Hall conductivity of Pt is markedly modulated by ionic gating, resulting in the gate-tunable ISHE^12^. In fact, the ISHE almost vanished in Pt grown on yttrium-iron-garnet (YIG) under a gate voltage of + 2 V using ionic gel as a gate material, where spin current was injected from the YIG under its ferromagnetic resonance (FMR). Ionic gating has opened a wide variety of novel physics, such as superconductivity in insulating oxide^13^ and transition metal dichalcogenides (TMD)^14,15^, chiral light emission from monolayer TMD^16^, and tunable Curie temperature (magnetization) in ultrathin Co^17^. These great achievements were attributed to the potential of ionic gating to accumulate very dense charges by the formation of an extraordinarily thin electric double layer. The physics behind the gate-tunable ISHE is the formation of nanometer-thick Pt (ultrathin Pt, with a thickness of ca. 2 nm) and Fermi level shifting due to the sufficient accumulation of electrons at the ultrathin Pt by ionic gating. Ultrathin Pt is considerably resistive, and its spin Hall mechanism is governed by the intrinsic mechanism, where the spin Hall conductivity *σ*_SH_ is dependent on the conductivity *σ* (*σ*_SH_ ~ *σ*^2^)^18^. The intrinsic spin Hall regime is governed by inter-*d*-band excitation, and the density of states of the *d*-orbital in Pt rapidly diminishes above the Fermi level^19^. Indeed, first-principle calculations revealed that the spin Hall conductivity of Pt decreased above the Fermi level^20^. A positive gate voltage application enabling electron accumulation gives rise to an upshift of the Fermi level from the intrinsic one in Pt, which allows suppression of the spin Hall conductivity, i.e., suppression of the ISHE.

Given that the SHE and the ISHE are interconnected by Onsager reciprocity, detection of the reciprocal effect of the abovementioned gate-tunable ISHE in ultrathin Pt, i.e., a gate-tunable SHE, can be expected. In a previous work^12^, spin current was injected from YIG to Pt by spin pumping, and the spin current was converted to charge current by the ISHE, which was gate-tunable, resulting in the detection of gate-tunable electromotive forces. Thus, one of the simplest reciprocal experiments to detect the gate-tunable SHE is planned as follows: an AC electric current flowing in ultrathin Pt allows generation of AC spin current due to the SHE. The generated AC spin current propagates into the adjacent ferromagnet and provides spin-torque to the ferromagnet, yielding spin-torque FMR (ST-FMR). The magnitude of the SHE can be suppressed by positive ionic gating and the ST-FMR is tunable, i.e., gate-tunable SOT. Consequently, the Gilbert damping parameter *α* of the ferromagnet can be tuned by ionic gating because the *σ*_SH_ of the ultrathin Pt is suppressed. Within this scheme, YIG is not appropriate as an adjacent material for the ST-FMR because YIG is a ferrimagnetic insulator and no electron conduction can flow in YIG, resulting in the no ST-FMR signal which is originated from the anisotropic magnetoresistance of a ferromagnet adjacent to Pt. Here, in this study, we demonstrate gate-tunable SOT as manifestation of Onsager reciprocity in spin-charge interconversion by using a Pt/Ni_80_Fe_20_ (Py) bilayer film. The magnitude of the SOT is measured as the change in *α* of Py in ST-FMR measurements. The modulation of the ST-FMR signal in our ST-FMR device by gating indicates the representation of the reciprocal effect of gate-tunable ISHE in Pt as studied in the previous work^12^.

## Result

**Figure 1:**
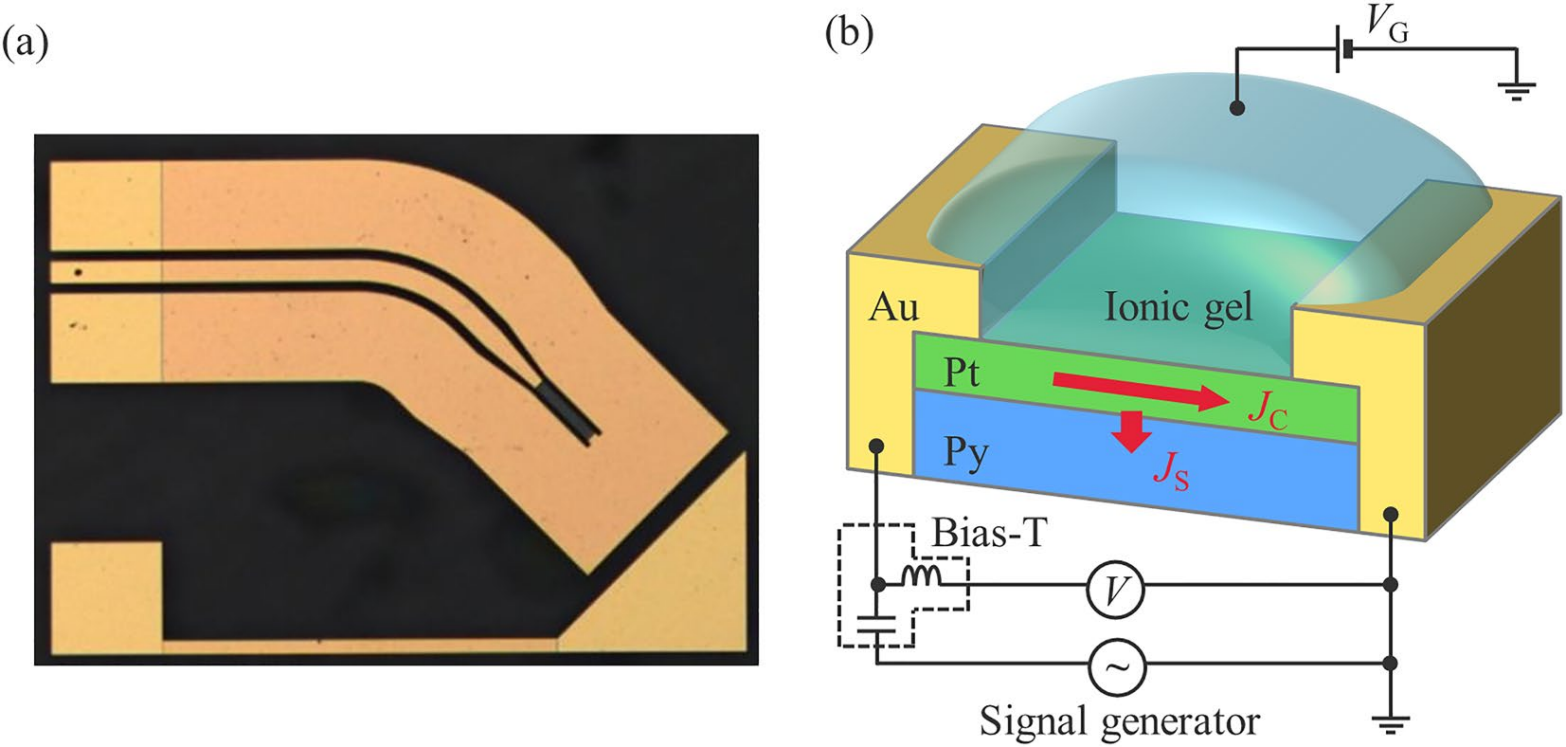
Structure of the gate-tunable ST-FMR device consisting of a Pt/Py bilayer. (**a**) Optical microscopic image of the ST-FMR device. (**b**) Schematic of the ST-FMR device and diagram of the ST-FMR measurement setup. Ultrathin Pt (1.2, 1.4 and 1.8 nm) is grown on Py (3 nm), and an ionic gel is coated onto the Pt. Gate voltages are applied via the ionic gel, enabling charge accumulation in the Pt. The experimental set-up measures the DC voltage produced across the device when applying an AC electric current, *J*_C_. The SHE in the Pt is modulated by gating, resulting in the modulation of the generated spin current, *J*_S_, by the SHE.

**Figure 2:**
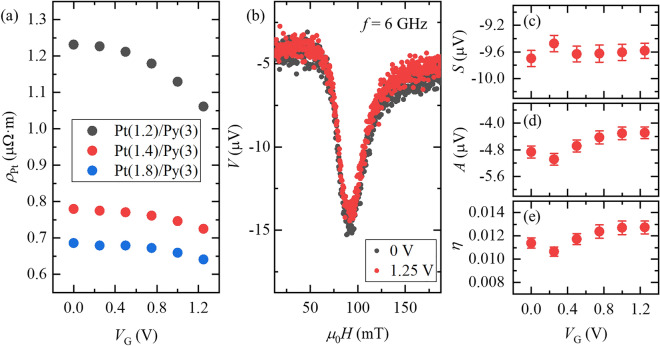
Gate dependence of the resistivity and ST-FMR signals of ultrathin Pt on Py. (**a**) Gate voltage *V*_G_ dependences of Pt resistivity *ρ*_Pt_ in three devices with different Pt thicknesses are displayed. All values in parentheses in the caption are in nm. The resistivity of Pt becomes greater as the thickness of Pt becomes smaller. The Pt resistivity decreased as a positive gate voltage was applied, which is due to charge accumulation by gating. (**b**) ST-FMR spectra of the Pt(1.2 nm)/Py(3 nm) device at gate voltages of 0 V (black closed circles) and 1.25 V (red closed circles). The frequency, *f*, was set to 6 GHz. Gate voltage dependence of (**c**) the symmetric component, *S*, (**d**) the anti-symmetric component, *A* and (**e**) spin-conversion efficiency, *η*.

Figure 1a and b show an optical microscopic image and a measurement circuit of a gate-tunable SOT (ST-FMR) device consisting of Pt/Py. To clarify how ionic gating modulates the resistivities of the Pt/Py devices, the gate voltage dependence of the resistivity of Pt (1.2, 1.4, and 1.8 nm) grown on Py (3 nm) was measured (see Fig. 2a). Gate voltages from 0 V to + 1.25 V were applied since the suppression of the ISHE in ultrathin Pt manifests itself only under a positive gate voltage^12^. Here, note that the gate voltage was limited at + 1.25 V because of a rapid increase in the gate leakage current above + 1.25 V, which hinders precise experiments (see Supplementary Information No. 1). The resistivity decreases as a function of gate voltage in all devices, and more importantly, modulation of Pt resistivity is the most prominent in the Pt (1.2 nm)/Py (3 nm) device. The decrease in Pt resistivity under a positive gate voltage is attributed to efficient charge accumulation, which also induces an upshift in the Fermi level. The modulation of Pt resistivity in the device is, in principle, consistent with that in the previous study^12^, i.e., thinner Pt revealed a stronger modulation of resistivity for the same gate voltage. Meanwhile, the gate voltage dependence of Pt resistivity is not substantially larger in Pt/Py than in Pt/YIG, which can be rationalized by the intrinsic carrier densities in the devices. In contrast to the previous study^12^, a metallic ferromagnet, not an insulator, is used in this study. To maintain equilibrium in the charge distribution in Py/Pt, carriers in Py flow into Pt in the formation of the bilayer because the carrier concentration of ultrathin Pt is considerably low (ca. 6 × 10^21^ cm^−3^)^12^. Thus, the carrier density of Pt on Py increases, which gives rise to a weak response in resistivity to the gate voltage resulting from the extrinsically modulated Fermi level in Pt. In a simple calculation using the Drude model, the modulation of the resistivity of 1.2 nm-thick Pt on Py (3 nm) was calculated to be 18% by the application of *V*_G_ =  + 1.25 V, which is equivalent to that measured here (12%, see also Supplementary Information No. 2).

Figure 2b–d show the ST-FMR signals from the Pt (1.2 nm)/Py (3 nm) device under *V*_G_ = 0 and 1.25 V and the gate voltage dependence of the symmetric components and anti-symmetric components of the ST-FMR signals, respectively, at the frequency *f* of 6 GHz. The ST-FMR signal is nicely deconvoluted by the conventional fitting function, and the results are fully reproducible in another device (see Supplementary Information No. 3 and No. 4). The ST-FMR signals are nicely seen at ca. 90 mT, which is ascribed to the fact that the AC electric current flowing in the 1.2 nm-thick Pt generates AC spin current and the AC spin current provides sufficient spin torque to Py to excite FMR. As seen in Fig. 2c and d, modulation of the symmetric component *S* and the anti-symmetric component *A* as a function of the gate voltage are indiscernible. Here, *S* is related to the spin current density *J*s generated by the spin Hall effect of Pt, whereas *A* is due to the sum of the Oersted field around the Pt and the field-like torque and is related to the charge current density *J*c^21^. Thus, the spin-conversion efficiency *η*, the ratio of spin current to charge current densities, also slightly changes with the gate voltage (see Fig. 2e). Without taking the spin-memory loss (SML) into account, *η* is described as,

1 $$\eta = \frac{{J_{{\text{S}}} }}{{J_{{\text{C}}} }} = \frac{S}{A}\frac{{e\mu_{0} M_{{\text{S}}} t_{{{\text{Py}}}} t_{{{\text{Pt}}}} }}{\hbar }\sqrt {1 + \frac{{M_{{{\text{eff}}}} }}{{H_{{{\text{res}}}} }}}$$

where *t*_Py_ and *t*_Pt_ are the Py thickness and the Pt thickness, ℏ is the Dirac constant, *μ*_0_ is the vacuum permeability, *M*_S_ is the saturation magnetization of Py*, M*_eff_ is the effective saturation magnetization of Py and *H*_res_ is the resonance field.

**Figure 3:**
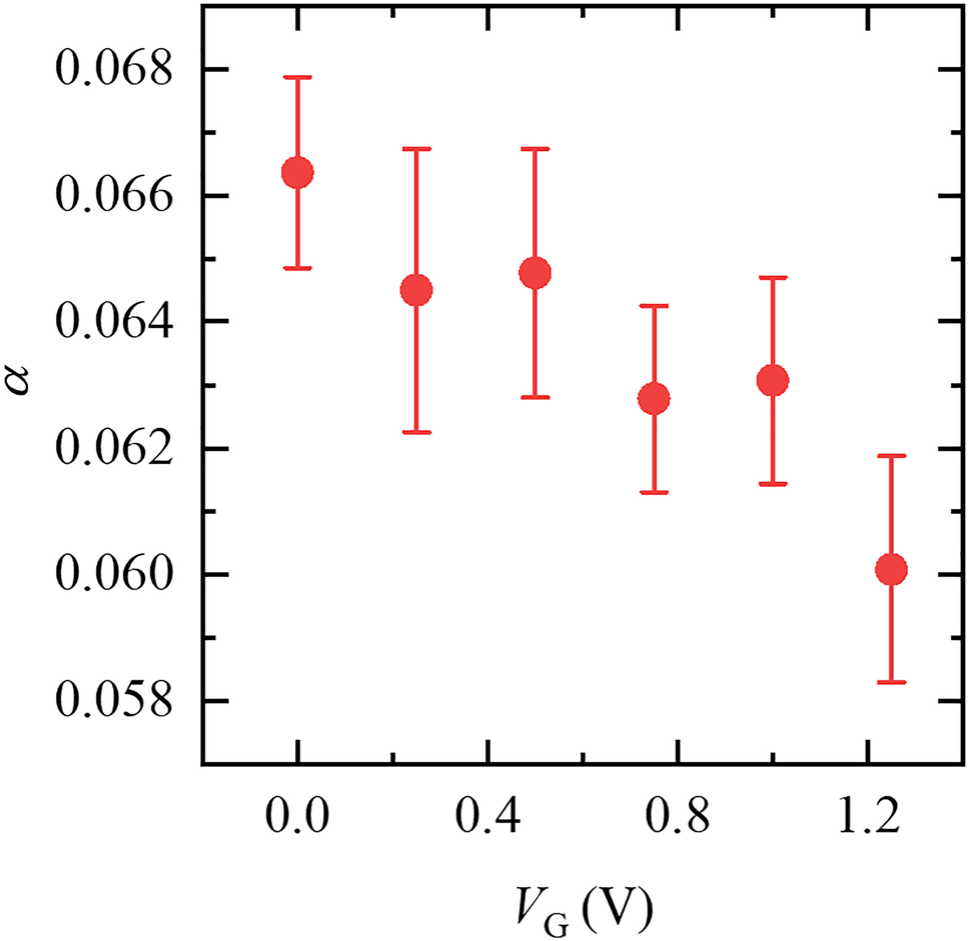
Gate-tunable Gilbert damping parameter, *α*. Gate voltage dependence of the Gilbert damping parameter, *α*. As a positive gate voltage is applied, *α* is decreased due to the suppression of the spin–orbit interaction in the gated Pt.

Nevertheless, the Gilbert damping parameter *α*, which is calculated from the frequency dependence of the half-width at half-maximum of the ST-FMR spectrum by using the relation, *Δ* = *Δ*_0_ + 2*παf* / *γ* (*Δ* and *Δ*_0_ are the half-width at half-maximum for non-zero *f* and *f* = 0 Hz, respectively, and *γ* is the gyromagnetic ratio), exhibits salient modulation under a positive gate voltage (see Fig. 3 and Supplementary Information No. 5). The Gilbert damping parameter *α* of a ferromagnet depends on the SOI of the adjacent nonmagnet, and *α* is smaller when the SOI of the adjacent nonmagnet is weaker. Hence, the smaller *α* under the greater positive gate voltage is ascribed to the weaker SOI of the gated Pt, i.e., the SHE (i.e., *σ*_SH_) in the 1.2 nm Pt is suppressed by positive gate voltages. Thus, this result demonstrates the reciprocal effect of the gate-tunable ISHE in ultrathin Pt^12^.

The spin Hall conductivity *σ*_SH_ is related to the spin Hall angle, which is described as the ratio of the spin current and charge current densities. Furthermore, the ratio of *S* and *A* in an ST-FMR spectrum is related to the spin Hall angle *η* (see Eq. 1). Meanwhile, *η* was indiscernible in the experiments under a positive gate voltage. As mentioned above, the symmetric components are attributed to the spin current density, and the anti-symmetric components are due to contributions from the Oersted field and the field-like torque. Thus, if merely the SOI of Pt in the Pt/Py device changes, only the symmetric component can be suppressed under gating, resulting in the suppression of *η *$\propto$* S*/*A*. However, this is not the case in reality, and it is hypothesized that a change in the field-like torque is why *η* is unchanged under gating.

The spin-conversion efficiency, *η*, can be expressed in terms of the damping-like-torque and field-like-torque efficiencies, *ξ*_DL_ and *ξ*_FL_, described as^22^:

2 $$\frac{1}{\eta } = \frac{1}{{\xi_{{{\text{DL}}}} }}\left( {1 + \frac{\hbar }{e}\frac{{\xi_{{{\text{FL}}}} }}{{\mu_{0} M_{{\text{S}}} t_{{{\text{Py}}}} t_{{{\text{Pt}}}} }}} \right)$$

**Figure 4:**
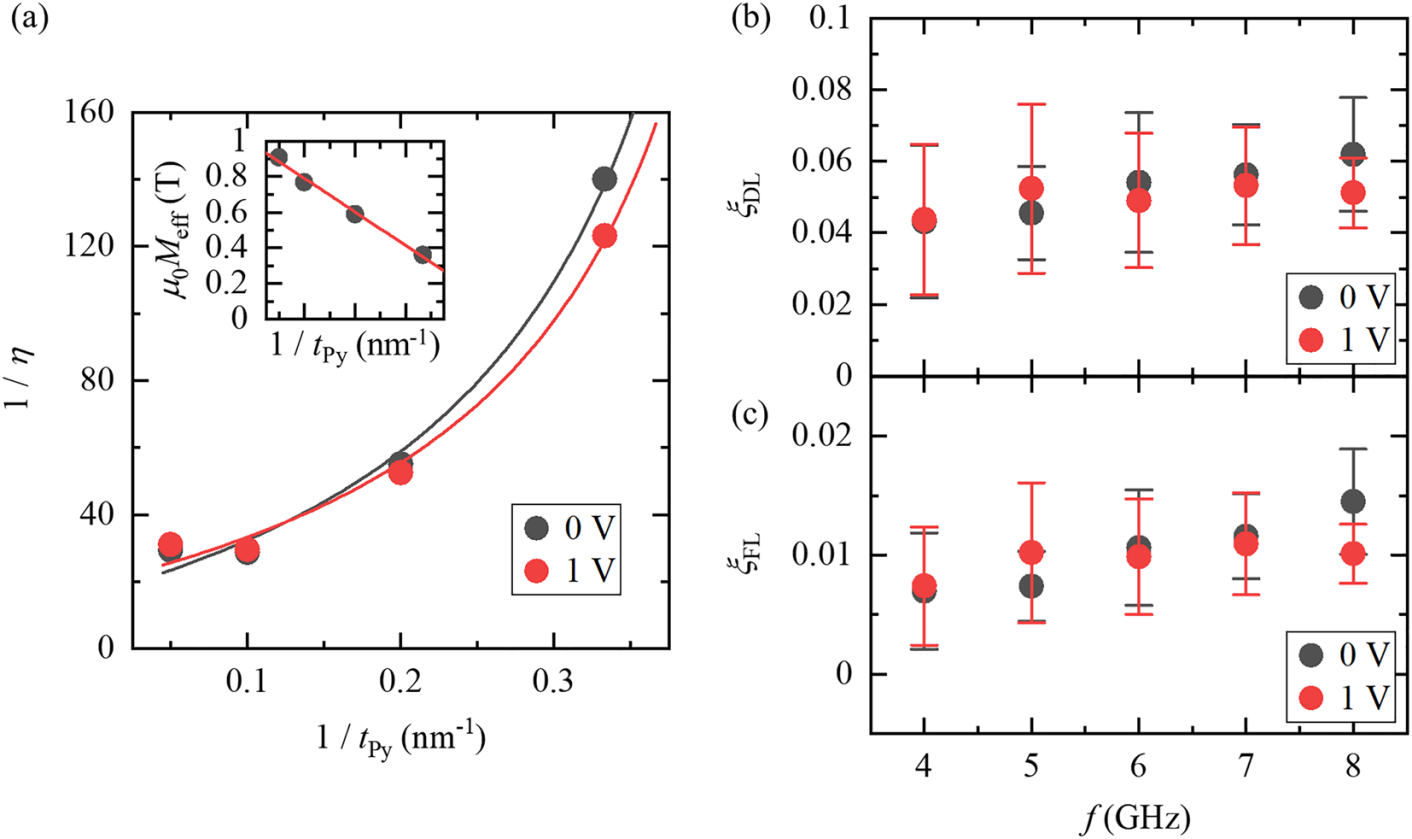
Estimation of damping-like torque and field-like torque. (**a**) Gate voltage dependence of the relationship between the inverse of the Py thickness *t*_Py_ and the spin-torque efficiency, *η*. The inset shows the Py thickness dependence of the saturation magnetization, *μ*_0_*M*_S_. Frequency and gate voltage dependences of (**b**) damping-like torque, *ξ*_DL_, and (**c**) field-like torque, *ξ*_FL_. The black and the red closed circles in all figures are the results at gate voltages of 0 and 1 V, respectively.

Then, *ξ*_DL_ and *ξ*_FL_ can be estimated via the *t*_Py_ dependence of *η* by postulating that both torques are independent of *t*_Py_. Here, *M*_S_ is inversely proportional to *t*_Py_, and *μ*_0_*M*_S_ ~ *μ*_0_*M*_eff_ = *a* / *t*_Py_ + *b*, where *a* and *b* are fitting parameters used to estimate the thickness-dependent *M*_S_^23,24^ (see the inset of Fig. 4a). Figure 4a shows the relationship between the inverse of *t*_Py_ and the inverse of *η* at *f* = 8 GHz with applied gate voltages *V*_G_ = 0 and 1 V. The results are nicely fitted by Eq. (2), and *ξ*_DL_ and *ξ*_FL_ are estimated to be 6.2 × 10^−2^ and 1.4 × 10^−2^ at *V*_G_ = 0 V and 5.1 × 10^−2^ and 1.0 × 10^−2^ at *V*_G_ = 1 V, respectively. Figure 4b and c show the frequency dependence of *ξ*_DL_ and *ξ*_FL_, respectively. The changes in *ξ*_DL_ and *ξ*_FL_ with the application of a gate voltage are indiscernible within the error bars, and the application of a gate voltage does not influence either the damping-like torque or field-like torque in this measurement.

## Discussion

Since there is no sizable change in either the damping-like or field-like torque by gating, we propose that gating modulates the spin-memory loss (SML)^25,26^, explaining the unchanged *S/A*. SML manifests in heterostructures consisting of a heavy metal and is attributed to the SOI of the heavy metal and interfacial disorder. SML gives rise to depolarization of the spin current propagating through the interface and is salient in Pt-based heterostructures. Indeed, the spin flip parameters, *δ*, in Co/Pt and Pt/Py are 0.9 and 0.63^27,28^, respectively, and are substantially larger than those of other *d*-electron-material-based heterostructures. Since the SOI is related to the SML as described above, modulation of the SOI by gating enables modulation of the SML. The right-hand side of Eq. (1), including the ratio *S*/*A*, is unchanged by gating in our experiments. However, in reality, the spin current generated by the SHE in Pt, *J*_S0_, does not fully flow into Py, and part of it dissipates at the interface due to the sizable SML. In fact, by considering SML, merely 53% of the spin current generated in Pt by the SHE flows into Py at *V*_G_ = 0 V (note that the spin flip parameter *δ* of Pt/Py is 0.63 and the spin depolarization at an interface in SML is described as [1 − exp(− *δ*)]). Hence, the transmitted spin current from Pt to Py at *V*_G_ = 0 V is equal to *J*_S0_exp(− 0.63). Meanwhile, under the application of *V*_G_ =  + 1.25 V, the charge current density flowing in Pt slightly increased (+ 14%; see the modulation of *ρ*_Pt_ shown in Fig. 2a), whereas the spin current density originally generated in Pt by the SHE at *V*_G_ =  + 1.25 V, *J*_S0_’, decreased to approximately 70% of that at *V*_G_ = 0 V, *J*_S0_ (see Fig. 4 of our previous report^12^). Since the ratio *S*/*A* is unchanged at *V*_G_ =  + 1.25 V, the spin flip parameter in Pt/Py at *V*_G_ =  + 1.25 V is estimated to be 0.14. The sizable suppression of *δ* by gating is due to suppression of the SOI in Pt by gating, given that SML originates from the SOI and the *δ* of a bilayer consisting of weak SOI metal is, for example, 0.017 for Pd/Py^28^ and 0.25 for Co/Cu^25^. Thus, the suppression of *δ* by gating also corroborates the gate-tunable SOI of Pt.

## Conclusion

In summary, we experimentally demonstrated the gate-tunable SHE of 1.2 nm-thick Pt grown on 3 nm-thick Py as manifestation of the gate-tunable SOI by employing ST-FMR and ionic gating techniques. The Gilbert damping parameter *α* was decreased by positive gating, which is due to the suppression of the SOI of ultrathin Pt by gating. Although the ratio of spin and charge currents in Pt was not substantially changed by gating, these changes are ascribed to the modulation of SML in Pt/Py. The suppression of the spin flip parameter *δ* in SML by gating was the other compelling result for the gate-tunable SOI in Pt. The results obtained in this study are a manifestation of the Onsager reciprocity between the SHE and the ISHE, and this study opens up a novel pathway to gate-tunable SOT devices and other gate-switchable spintronic devices.

## Methods

### Sample fabrication procedure

A Pt(*d* nm)/Py(*t* nm) bilayer (20 μm × 140 μm) was deposited on a MgO substrate by the lift-off process using electron-beam (EB) deposition and EB lithography. (Note that the Pt layer was on top.) The thickness *d* of the Pt layer was set to 1.2, 1.4 and 1.8 nm, and the thickness *t* of the Py layer was set to 3, 5, 10, and 15 nm. Then, a shortened coplanar waveguide was fabricated as the Pt/Py became the signal line. To suppress a leakage current during gate voltage application, PMMA/MMA resist was coated on the waveguide and developed to mask all parts of the device except for the Pt/Py, probe contacts, and side gate electrode. Finally, ionic gel was mounted on the device and the side electrode. The ionic gel was made by mixing DEME-TFSI ionic liquid (Kanto Chemical, Japan), PS-PMMA-PS polymer (Polymer Source, USA), and ethyl propionate (CH_3_CH_2_COOC_2_H_5_) in a weight ratio of 9.3:0.7:20. The sample for resistance measurement was fabricated by the same procedure on a SiO_2_(300 nm)/Si substrate.

### Measurement and analysis of the ST-FMR spectrum

An RF current with an input power of 10 mW (10 dBm) was applied in the longitudinal direction of the sample with an in-plane external magnetic field *H*_ext_ at an angle of *θ* = 45° with respect to the longitudinal direction. The frequency of the RF current was set to 4, 6 and 8 GHz. All measurements were carried out at room temperature.

FMR is caused by the Oersted field due to a charge current in the Pt layer and a spin current generated by the spin Hall effect in Pt. The field-induced FMR spectrum has an anti-symmetric component in an output voltage at the resonance field. Meanwhile, the spin current due to the spin Hall effect in the Pt layer is injected into the adjacent Py layer and excites a spin-current-induced FMR, which has a symmetric component in the output voltage. The mixed FMR of these two components can be detected as a DC voltage via the rectification effect, which can be expressed as^21^, *V* = *C*[*AF*_A_(*H*_ext_) − *SF*_S_(*H*_ext_)], which is the fitting function used in this study. Here, *C* is a coefficient related to the anisotropic magnetoresistance, *F*_S_(*H*_ext_) = *Γ*^2^/[(*H*_ext_ − *H*_res_)^2^ + *Γ*^2^] is a symmetric Lorentzian with a resonance field *H*_res_ with *Γ* being the half width at half maximum of the ST-FMR spectrum, and *F*_A_(*H*_ext_) = *F*_S_(*H*_ext_) × [(*H*_ext_ − *H*_res_)/*Γ*] is an anti-symmetric Lorentzian. The symmetric component *S* is related to the spin current density *J*_S_ generated by the spin Hall effect of Pt, whereas the asymmetric component *A* is due to the sum of the Oersted field around the Pt and the field-like torque and is related to the charge current density *J*_C_.

## Supplementary Information

Supplementary Information.
