# Supplementary material for: Modulation of spin-torque ferromagnetic resonance with a nanometer-thick platinum by ionic gating
Source: Sci Rep. 2021 Nov 5;11:21779. doi: 10.1038/s41598-021-01310-6 (PMC8571418; doi:10.1038/s41598-021-01310-6)
Supplement: Supplementary file 1 — Supplementary Information. [file 41598_2021_1310_MOESM1_ESM.pdf]

Supplementary Information

Modulation of spin-torque ferromagnetic resonance with a nanometer-thick platinum by ionic gating

R. Ohshima[*,†], Y. Kohsaka[*], Y. Ando, T. Shinjo, and M. Shiraishi[†]

Department of Electronic Science and Engineering, Kyoto University, Nishikyo-ku, Kyoto 615-8510, Japan

No. 1 Gate leakage current under gating

We monitored the gate leakage current during the spin-torque ferromagnetic resonance (ST-FMR) under gating. Figure S1 shows the gate voltage dependence of the gate leakage current in the ST-FMR device discussed in the main text. A rapid increase in the leakage current can be observed when gate voltage is greater than 1.5 V. Meanwhile, the leakage current is sufficiently suppressed below V_G = +1.25 V, and the experiments for the modulation of the Gilbert damping parameter α, was carried out below the gate voltage of +1.25 V.

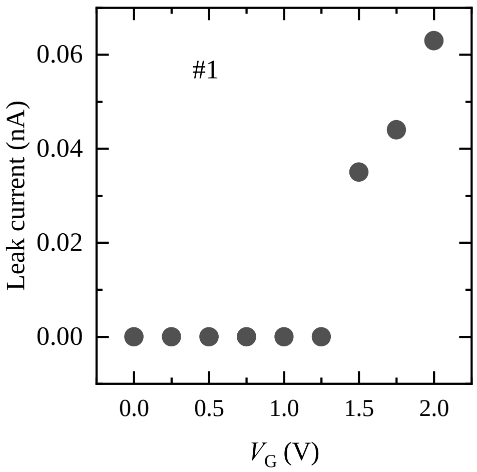

Fig. S1. The gate voltage dependence of the gate leakage current of the Pt(1.2 nm)/Py(3 nm) ST-FMR device discussed in the main text.

No. 2 Estimation of the total carrier density in the Pt(1.2 nm)/Py(3 nm) bilayer film.

In our previous study [S1], the carrier density of the 2 nm-thick Pt film was estimated to be 6.0 × 10^{21} cm^{-3} from the thickness dependence of the Pt films and the model calculation described in ref. [S2]. We postulate the carrier density in such the ultrathin Pt films is not changed. From a simple Drude model, the carrier density of Py is estimated to be 4.8 × 10^{22} cm^{-3}, where the work function of Py is set to 4.8 eV [S3]. Given that the thicknesses of Pt and Py, the sheet carrier density of the Pt/Py bilayer is estimated to be 1.1 × 10^{15} cm^{-2}. An accumulated charge density under ionic gating used in our setup was estimated to be 2.0×10^{14} cm^{-2}/V [S1], and thus, the charge density at V_G = 1.25 V is estimated to be 2.5 × 10^{14} cm^{-2}, resulting in ca. 18% modulation of the resistivity of Pt (1.2 nm) on Py (3 nm). As shown in Fig. 2 of the main text, the modulation of Pt (1.2 nm) by the application of V_G = 1.25 V was measured to be ca. 12%, which is equivalent to that obtained by the model calculation.

No. 3 Peak deconvolution of the ST-FMR signal

Figure S2 shows the ST-FMR signal shown in the Fig. 2(b) of the main text and the result of the peak deconvolution. The detail of the deconvolution is described in Methods in the main text. As seen, the ST-FMR signal is nicely deconvoluted, and the magnitudes of the symmetric (S) and anti-symmetric (A) components are quantitatively characterized.

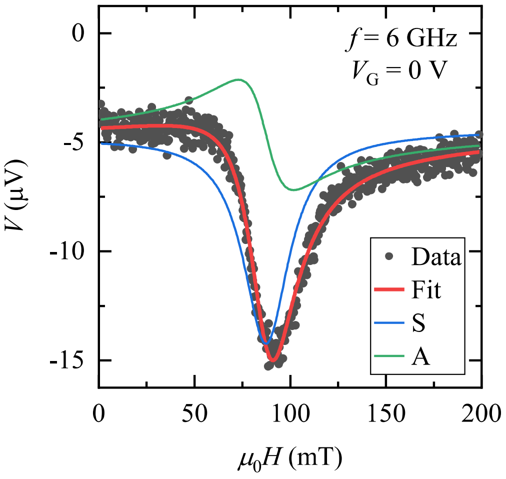

Fig. S2 The ST-FMR signal from the Pt(1.2 nm)/Py(3 nm) device at f = 6 GHz and V_G = 0 V. The red line indicates the fitting result of the experimental data (black filled-in circle). The symmetric (S) and anti-symmetric (A) components are shown in the blue and green lines, respectively.

No. 4 Reproducibility of the gate-tunable spin torque

We prepared other devices with same structure and implemented the same experiment to confirm the reproducibility of the gate-tunable spin torque. Figure S3 shows the results of the gate voltage dependences of the ST-FMR signals, the S and A components, the spin-torque efficiency η, and α. Figure S4 shows the gate voltage dependence of the gate leakage current of the same device. Unlike the device discussed in the main text, the gate leakage current gradually increases as a function of gate voltages, and is sizable when the gate voltage was +1.25 V.

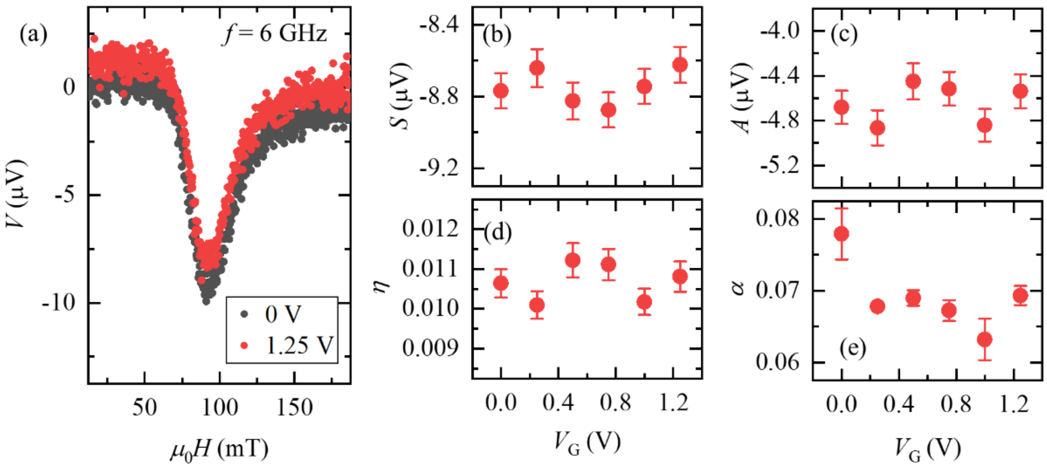

Fig. S3 (a) ST-FMR spectra of the other Pt(1.2 nm)/Py(3 nm) device at the gate voltage of 0 V (black closed circles) and 1.25 V (red closed circles). The frequency, f, was set to 6 GHz. The gate voltage dependence of (b) the S component, S, (c) the A component, A, (d) the spin-torque efficiency, η, and (e) the Gilbert damping parameter, α.

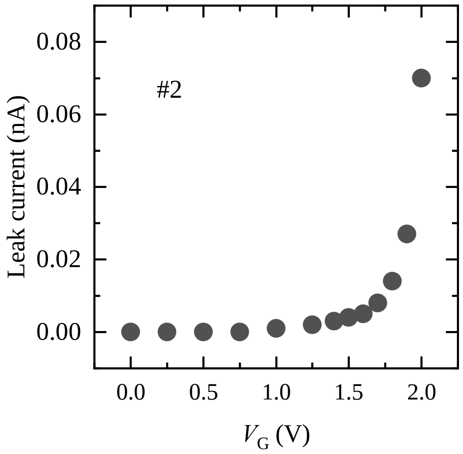

Fig. S4 Gate voltage dependence of the gate leakage current of the same ST-FMR device discussed in the Supplemental Information.

No. 5 The frequency dependence of the half-width at half-maximum of the ST-FMR spectrum

To quantitatively estimate the gate voltage dependence of the Gilbert damping parameter, α, the frequency dependence of the half-width at half-maximum of the ST-FMR spectrum was measured. Fig. S5 shows ST-FMR signals measured at V_G = 0 V and the frequency, f, is set to 4, 6, and 8 GHz, respectively. Each signal was fitted via the equation for the mixed FMR spectrum (see Methods in the main text) to obtain the resonance field, H_{res}, and the half-width at half-maximum, Δ. The frequency dependences of each parameter are given as the Kittel equation [S4] and the Gilbert damping equation[S5] : $f = g\mu_B\mu_0(H_{res}(H_{res} - M_{eff}))^{0.5} / 2\pi\hbar$ and $\Delta = \Delta_0 + 2\pi\hbar\alpha f / g\mu_B$, where $\hbar$ is the Dirac constant, g is the g-factor, μ_B is the Bohr magneton, μ_0 is the vacuum permeability, M_{eff} is the effective saturation magnetization of Py, and α is the Gilbert damping parameter. Δ_0 is the Δ at f = 0 GHz, corresponding to the frequency independent scattering process. Fig. S6 and S7 are the frequency dependences of H_{res} and Δ measured with gate voltages from 0 V to +1.25 V and their fitting results. As seen, each frequency dependence was well fitted with the equations.

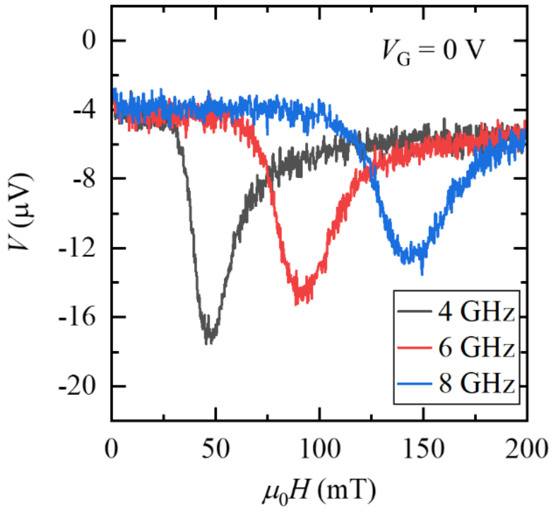

Fig. S5 The ST-FMR signals measured at V_G = 0 V and f is set to 4, 6, and 8 GHz, respectively.

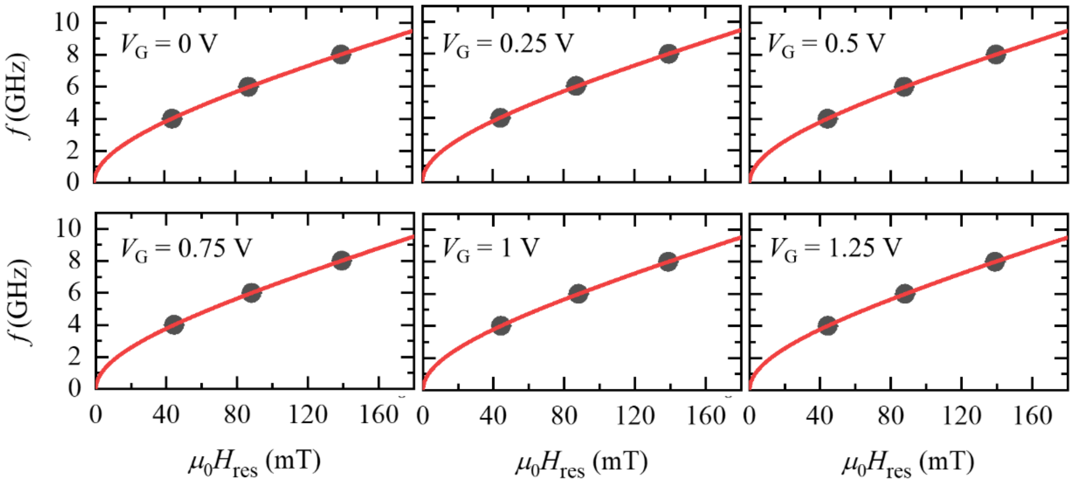

Fig. S6 The frequency dependence of the resonance field of the ST-FMR signals H_{res} with various gate voltages. The red lines indicate the fitting result of the experimental data (black filled-in circle).

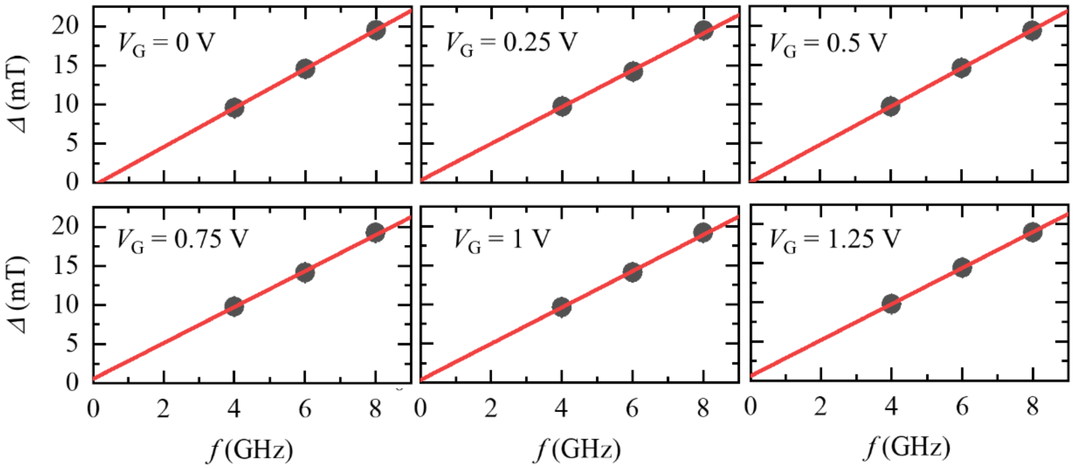

Fig. S7 The frequency dependence of the half width at half maximum of the ST-FMR signals Δ with various gate voltages. The red lines indicate the fitting result of the experimental data (black filled-in circle).

No. 6 ST-FMR spectra in bipolar setup

Figure S8 shows the ST-FMR spectra in bipolar setup; the magnetic field was applied along 45 degrees and 225 degrees to the rf current direction. The spectra have a symmetric shape, indicating there are negligible spurious effects such as the thermal effect. Fittings are performed by the method in the main text.

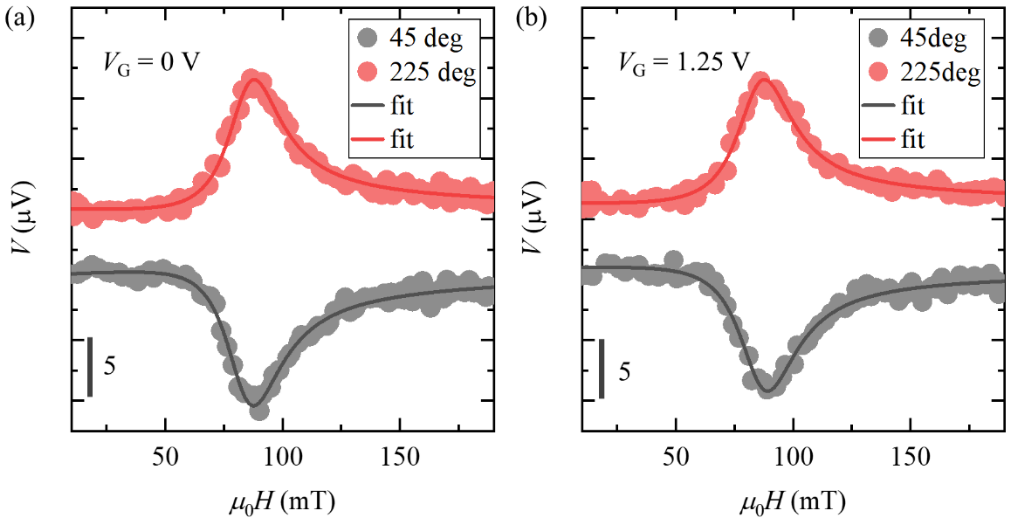

Fig. S8 Bipolar ST-FMR spectra at (a) $V_G = 0$ V and (b) $V_G = 1.25$ V. The spectra have a symmetric shape for both gate voltages.

References

[S1] Dushenko, S., Hokazono, M., Nakamura, K., Ando, Y., Shinjo, T. & Shiraishi, M. Tunable inverse spin Hall effect in nanometer-thick platinum films by ionic gating. *Nat. Commun.* **9**, 3118 (2018).

[S2] Fan, P., Yi, K., Shao, J.-D. & Fan, Z.-X. Electrical transport in metallic films. *J. Appl. Phys.* **95**, 2527–2531 (2004).

[S3] Wang, W., Liu, Y., Tang, L., Jin, Y., Zhao, T. & Xiu, F. Controllable Schottky barriers between MoS_2 and permalloy. *Sci. Rep.* **4**, 6928 (2014).

[S4] Ando, K. & Saitoh, E. Inverse spin-Hall effect in palladium at room temperature. *J. Appl. Phys.* **108**, 113925 (2010).

[S5] Nembach, H. T., Silva, T. J., Shaw, J. M., Schneider, M. L., Carey, M. J., Maat, S. & Childress, J. R. Perpendicular ferromagnetic resonance measurements of damping and Landé g-factor in sputtered $(Co_2Mn)_{1-x}Ge_x$ thin films. *Phys. Rev. B* **84**, 054424 (2011).
